# Patient-perceived treatment burden of tuberculosis treatment

**DOI:** 10.1371/journal.pone.0241124

**Published:** 2020-10-22

**Authors:** Natasha C. H. Ting, Nicole El-Turk, Michael S. H. Chou, Claudia C. Dobler

**Affiliations:** 1 Department of Respiratory and Sleep Medicine, Liverpool Hospital, Sydney, NSW, Australia; 2 South Western Sydney Clinical School, University of New South Wales, Sydney, NSW, Australia; 3 Institute for Evidence-Based Healthcare, Bond University and Gold Coast University Hospital, QLD, Australia; ESIC Medical College & PGIMSR, INDIA

## Abstract

**Background:**

Treatment for tuberculosis lasts for a minimum of 6 months. The treatment burden experienced by patients in a low-incidence setting where directly observed therapy is the standard of care is not well-known.

**Methods:**

Patients receiving tuberculosis treatment through the chest clinic at a tertiary hospital in Sydney, Australia, participated in a semi-structured interview. The interviews explored the treatment burden experienced by patients and possible solutions to ameliorate this burden. Interviews were conducted until data saturation was achieved. They were recorded, transcribed and analysed using NVivo 12 software.

**Results:**

Twenty participants (80% male, mean age 40 years) with pulmonary (n = 13) and extra-pulmonary (n = 7) tuberculosis were interviewed. Participants experienced healthcare, financial, social and medication burdens along with lifestyle changes due to treatment. Medication intake was challenging due to the high number of pills, and 55% (n = 11) of patients experienced fatigue amongst other side effects. Patients found clinic-based directly observed therapy inconvenient, especially those working and/or studying. Suggestions to lessen treatment burden included reducing medication burden and better access to health services.

**Conclusion:**

Tuberculosis treatment is associated with substantial treatment burden for patients. Measures to reduce treatment burden including alternative treatment delivery methods which are more accommodating to patients than clinic-based directly observed therapy, such as video directly observed therapy or partially self -administered treatment, should be considered on a case-by-case basis.

## Introduction

First-line treatment for drug-sensitive tuberculosis (TB) as outlined by the World Health Organization (WHO) involves a combination of isoniazid, rifampicin, pyrazinamide and ethambutol for a minimum of 6 months [1]. WHO recommends that these medications are administered through directly observed therapy (DOT), where patients’ consumption of medications is observed by trained personnel (usually a healthcare worker) [2]. However, a Cochrane review concluded that DOT does not improve TB treatment completion and cure compared with self-administered treatment [3].

Treatment burden is the workload that a patient must manage to take care of their health and its impact on the patient’s daily life [4]. Examples of treatment burden include scheduling appointments, travelling to appointments or managing medications. Time-consuming treatments in chronic diseases without adequate support of the patient or communication between healthcare providers can negatively impact clinical outcomes [5–7]. Medication [8, 9], social [9, 10] and financial burdens [11] are commonly experienced by patients with TB. Previous interview studies have demonstrated that there is a ‘pill burden’ caused by the many tablets that patients must consume and related medication side effects [8, 9]. This burden substantially increases in patients with multi-drug resistant TB with an increased treatment timeframe (up to 24 months) and the inclusion of injectable medications [12]. Socially, receiving TB treatment (particularly through DOT) can also worsen real or perceived stigma associated with TB, as patients might fear that people around them will learn about their disease when they know that they are attending the TB clinic. Patients may therefore avoid visiting treatment clinics and be reluctant to take medication [10]. Interlinked with medication and social burden is the impact on a patient’s finances. Patients are required to travel to a central clinic for clinic-based DOT, which comes at a direct cost to the patient (public transport, fuel, parking etc.) as well as indirect or opportunity costs (e.g. unpaid leave) [8, 11].

Given the lack of evidence that clinic-based DOT improves TB treatment completion and cure [3] but anecdotal experience of a significant treatment burden associated with DOT in our patients, we aimed to explore the treatment burden associated with TB treatment in a low-incidence setting for TB, where clinic-based and video DOT are commonly used, and to explore ways to alleviate treatment burden. The gathered data can inform patient-centred health service delivery and health policies for TB treatment.

## Methods

### Study design, setting and participants

We used semi-structured interviews to obtain an in-depth understanding of patient experiences of treatment burden associated with TB treatment. The study was conducted at the chest clinic of Liverpool Hospital in South Western Sydney, New South Wales, Australia, which serves a multicultural community. The TB incidence rate of 9.02 per 100,000 in this area is one of the highest in the state of New South Wales which had an incidence of TB of 6.4 per 100,000 in 2018 [13]. In the state of New South Wales, Australia, clinic-based DOT has been the standard of TB treatment until recently. However, chest clinic nurses deliver treatment to TB patients in the community while patients are infectious or if they are elderly and impaired. All TB services (including medications for the disease and for treatment of side-effects) are provided free of charge, through local chest clinics affiliated with public hospitals [14]. More recently, accelerated by the COVID-19 pandemic, video DOT (where supervision is delivered digitally) [15] and a combination of clinic-based DOT (e.g. once every two weeks) and self-administered therapy (SAT) are increasingly used as alternatives to standalone clinic-based DOT. These alternatives provide flexibility and privacy whilst saving time and direct costs to the patient [16].

Eligible study participants were (1) undergoing DOT, a combination of DOT and SAT or video DOT for active pulmonary or extra-pulmonary TB, or had completed treatment in the past 12 months, (2) were at least 18 years of age and (3) could communicate in English without the need for an interpreter. Potential participants were identified through the chest clinic’s TB notification records and recruited in the clinic or over the phone. Purposive sampling was used to recruit patients with different characteristics (age, sex, treatment type and duration). Participants were provided with a participant information leaflet and written or verbal consent (for phone interviews) was obtained prior to conducting interviews. Ethical approval for this study was obtained from the South Western Sydney Local Health District Human Research and Ethics Committee.

### Data collection

A preliminary TB specific treatment burden framework which was informed by a taxonomy of the burden of treatment [17], a literature review and input from TB experts was used to create an interview guide specific to this study. This guide was piloted in 3 interviews. A final revised interview guide was used for all other interviews (S1 File). Interviews were conducted by one of the investigators (NCHT) until data saturation was achieved. Interviews were performed either in person (i.e. face-to-face) or over the telephone, both within a clinic room. These were voice-recorded, transcribed and uploaded into the qualitative analysis software NVivo 12. Pseudonyms were then assigned to each participant.

### Data analysis

A deductive-inductive approach was used to code for themes using NVivo 12. Information supporting the preliminary framework was searched for and coded into pre-determined categories (nodes) and sub-categories (sub-nodes) during the first round of narrative coding. New information not included in the framework was noted and through discussion with study investigators, new categories were created during the coding process. A second round of coding was then performed on all interviews to identify themes belonging in the new categories. The final TB treatment burden framework was created to include updated categories. One of the investigators (NCHT) coded all interviews. The coding framework was reviewed and adjusted after the first two interviews by the senior investigator (CCD). Two other investigators (NET, MSHC) independently coded four randomly selected interview transcripts for quality assurance purposes. Any differences were discussed and reviewed.

## Results

Of 32 patients who initially agreed to be interviewed, three later withdrew and nine could not be further contacted for an interview. Overall, 20 participants were interviewed with 15 in-person and five phone interviews. Interview times ranged from 18 to 52 minutes, with a mean of 27 minutes. Table 1 summarises patient and treatment characteristics.

**Table 1:** Participant information.

Characteristic	n	%
**Gender**		
*Male*	16	80
*Female*	4	20
**Age in years**		
*Range*	18 to 70	
*Mean*	40	-
**Country of birth**		
*Australia*	3	15
*Bangladesh*	1	5
*India*	4	20
*Indonesia*	2	10
*Myanmar*	1	5
*Nepal*	2	10
*Philippines*	2	10
*Sierra Leone*	1	5
*Vietnam*	3	15
*Zimbabwe*	1	5
**Type of tuberculosis**		
*Pulmonary*	13	65
*Extra-pulmonary*	7	35
**Current treatment status**		
*Intensive phase (<2 months) or isolation*	9	45
*Continuous phase (>2 months)*	10	50
*Completed treatment*	1	5
**Employment status**		
*Retired*	3	15
*Unemployed*	2	10
*Employed and/or studying*	15	75
**Form of therapy**		
*Clinic based directly observed therapy*	14	70
*Video directly observed therapy*	5	25
*Other*^*a*^	1	5
**Form of transport used to the TB clinic**		
*Public transport*	6	30
*Car*	14	70
**Reported number of tablets per daily dosage (excluding completed patient)**		
*Less than 10 tablets*	10	52.6
*More than 10 tablets*	9	47.4

^a^ Patient previously was on clinic-based DOT but moved onto an altered treatment regimen of visiting clinic once-fortnightly and had 13 days take home supply.

### Treatment burden discovered

**Fig 1:**
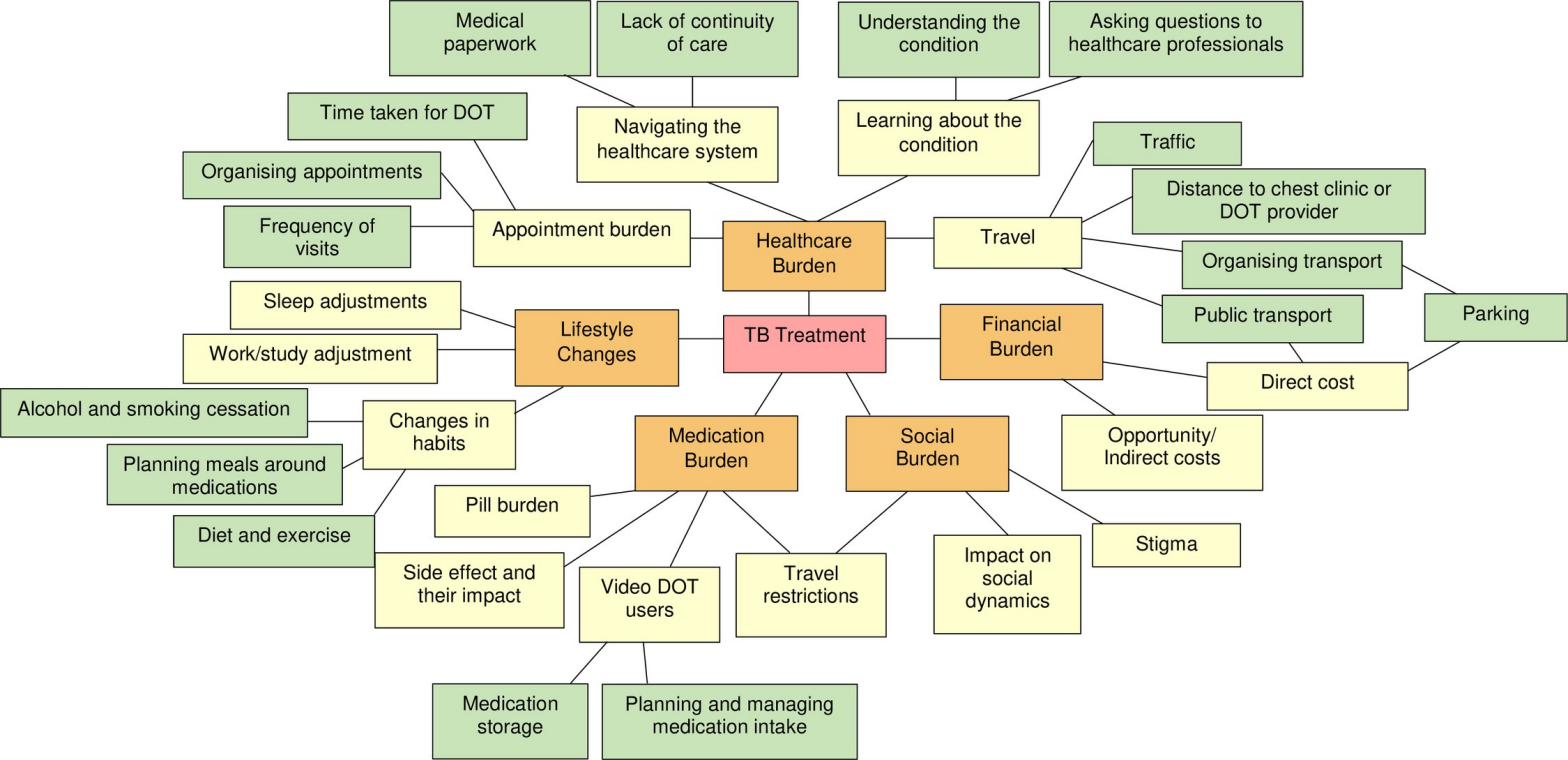
Treatment burden framework for tuberculosis. Red: main theme; orange: main categories of treatment burden; yellow: sub-themes; green: specific forms of treatment burden.

Thirteen types of treatment burden were identified by patients: direct costs, indirect costs, appointment burden, lack of continuity of care, medical paperwork, travel burden, travel restrictions, impact on social dynamics, pill burden, medication side effects, adjustment of work and study, adjustment of sleep and change in dietary and other habits (Table 2). These were further summarised under five broad categories of financial burden, healthcare burden, medication burden, social burden and lifestyle changes. Suggestions made to ameliorate treatment burden were summarised under two categories: better provision of health services and access, and a less burdensome treatment regimen. The preliminary treatment burden framework was revised based on interview findings and a final framework was produced (Fig 1).

**Table 2:** Treatment burden perceived by patients during tuberculosis treatment.

Healthcare Burden					Financial Burden		Social Burden		Medication Burden		Lifestyle Changes		
	Appointment burden	Medical paperwork	Lack of continuity of care	Travel to clinic	Direct Cost	Indirect Costs	Impact on social dynamics	Travel restrictions	Medication intake	Side effects	Planning meals	Work and study adjustments	Sleep adjustments
Anna	+	+	-	+	-	+	+	-	-	+	+	+	+
Aarush	-	-	-	-	-	+	-	+	+	+	+	+	+
Amod	+	+	-	+	+	**+**	+	+	+	+	+	+	+
Edward	+	-	-	+	+	+	+	+	+	+	+	+	-
Isabella	+	-	-	+	-	-	+	+	+	+	+	+	+
Jerod	+	+	+	+	+	+	+	-	-	+	+	+	-
John	+	+	-	+	-	-	+	-	-	+	+	-	-
Joseph	-	-	-	+	-	-	-	-	-	+	-	+	-
Khang	-	-	-	+	-	-	-	-	-	-	-	-	-
Manny	-	-	-	-	-	-	+	-	-	+	-		-
Mahir	+	-	-	+	+	+	-	+	+	+	-	+	-
Masud	+	-	-	+	+	-	+	-	-	+	+	+	+
Minh	+	-	-	+	+	-	+	-	-	+	+	-	-
Nandin	+	+	+	+	-	-	+	-	-	-	+	+	-
Rina	-	-	-	-	+	-	+	-	+	-	+	+	-
Raahul	+	-	-	+	-	-	-	-	+	+	+	+	-
Saachi	-	-	-	+	+	+	+	+	-	+	-	+	-
Saharsh	+	-	+	+	-	+		+	-	+	+	+	-
Tinh	-	-	-	-	-	+		+	+	-	+		-
Thiri	-	-		+	-	-			-	+	+		-

+, indicates burden; -, indicates no burden; an empty field indicates that burden was not discussed or not applicable.

### Financial burden

#### Direct costs

Fifteen participants incurred travel costs associated with chest clinic visits. Parking costs ranged from $0 up to $20 per visit, average petrol costs were an estimated $10 per visit whilst public transport costs ranged from $3 to $7 per visit. Costs depended on distance to clinic, visitation frequency, type of petrol used and the car model. Some participants identified these costs as being burdensome. One participant described his health as more important than financial hardship. Two participants received a fine for parking overtime in a designated patient parking spot while attending the TB clinic, which added to their financial burden.

“When they first told me I was gonna [sic] have to go in everyday, yea, that would have been a nightmare paying for parking and petrol and not being able to work”–Anna, working mother with young children

#### Indirect costs

Time taken off work to attend chest clinic DOT resulting in loss of income affected more than half of participants, with 9 experiencing a financial burden as a result. Indirect costs affected most working participants regardless of whether they were paid an hourly salary or were on a fixed income. Those with a fixed salary often had to take more sick leave to attend appointments than the amount they were entitled to, and therefore lost income as well.

“*When you’re interrupted for 2 hours everyday*, *it interrupts everything…2 hours I cannot work…one fourth of my time*. *Definitely it affects my income”–Mahir*, *health professional*

### Healthcare burden

#### Appointment burden

Patients visited the chest clinic in person (for DOT or doctor visits) or attended scheduled video appointments for video DOT. Visitation frequency ranged from daily to thrice weekly; three participants visited less than once a week and one received community-based DOT through TB clinic staff. Two participants recalled waiting at least an hour for their doctor appointment.

Frequent appointments were inconvenient and disruptive, causing patients having to rearrange work and study commitments and forgo social events or travel. Co-ordination between specialist appointments for different health issues was also a burden, due to limited timeslots and appointment clashes. Rescheduling appointments often caused further delay.

“Probably the most hardest part of this treatment is to come in…you need an extra 3 hours in the bank to accommodate for this, it gets pretty inconvenient if you have to come every day”–Amod, student and part-time worker

#### Lack of continuity of care

Some patients who also saw a specialist for a condition other than TB experienced a lack of communication between doctors, making their care fragmented.

#### Medical formalities

Medical work-up (e.g. imaging, blood tests) and paperwork was sometimes required and a hassle to obtain. Some patients had to wait during prolonged periods when they required a medical certificate outside of a scheduled doctor’s appointment.

#### Travel to treatment centre

Patients commonly found travelling to the chest clinic for DOT inconvenient and time-consuming. A quarter of participants found their return trip stressful due to medication side effects. For some, daily travel to the TB clinic was very cumbersome because they lived far away.

“I literally would have to give up work and even then I’d still be on an hour and a half each way, that’s 3 hours round…so I’ve got to drive and I’ve got to be doing that everyday just to get medication.”–John, works in sales

Most participants drove to the TB clinic by car and experienced stress with traffic and parking. A small group of participants took public transport and all except one found travel time to be long or tiresome, with irregular transport schedules adding to this frustration.

“It’s a waste of traffic time, it’s a waste of my time, you’re burning petrol. So I travel 20 minutes but I’m not sure [about] people who have to travel longer distances…”–Saharsh, recently commenced DOT

### Medication burden

#### Pill burden

Patients on average had to take 10 tablets per daily dosage. The high number of tablets and the unpleasant taste made medication consumption difficult. Six participants found the medications hard to swallow, with one taking up to an hour to swallow his daily dose. Some required tablets to be crushed or opted for a liquid alternative where possible.

“When there was [sic] 4 drugs, it was a lot, like 16, 17 tablets a day. Just swallowing them is not an easy task and you still get nauseous after taking them”–Edward, TB patient

#### Side effects

Participants experienced at least one side effect with fatigue (n = 11) and nausea (n = 9) being most commonly reported. Other side effects included skin rash, heartburn, weight loss, loss of appetite, and numbness in arms or legs. Two patients had treatment paused due to severe side effects and required an adjusted treatment regimen.

“I do have symptoms of nausea and all that, all the time, even with like metoclopramide and also ondansetron…I still feel nauseous”–Isabella, TB patient

Fatigue was the main concern for patients, having an impact on their usual daily routine. Studying and working participants had to accommodate for the symptom by rearranging schedules. Participants generally found the side effects of treatment more tolerable as treatment progressed or as the number of tablets decreased over time.

“It really did affect me work wise and studying because if I were to take the medications, I just automatically become like the zombie…in some instances, I’ll just sleep…you really do feel really tired, so tired”–Jerod, student and employed worker

### Social burden

#### Impact on social dynamics

Some patients missed out on social events due to treatment appointments whilst others found it hard to socialize when they experienced side effects of TB treatment. Seven participants kept their treatment confidential except for sharing the information with close family and friends, and three participants felt stigmatised (e.g. avoided) when people knew that they were receiving treatment for TB. Four participants avoided contact with others after their diagnosis, being worried about infecting others.

#### Travel restrictions

For some, treatment restricted travel interstate or overseas for work and/or leisure, due to the need to take medications. One participant mentioned that the number of medications they would have to bring discouraged them from travelling. Other participants had no issues and did not feel that their treatment regimen restricted their ability to travel.

### Lifestyle changes

#### Adjustment in work and study

For workers and students, appointments and symptom management impacted on productivity levels. Students experienced difficulties in managing classes and study time, whilst workers experienced disruptions to work commitments. One patient felt burdensome to his colleagues as they were required to work around his treatment schedule.

“I had to go to the hospital every day, it was a bit challenging because I had to go to the hospital first and then go to work, and it had a significant impact on my work because it was quite stressful because both are equally important…”—Nandin, full-time worker

#### Adjustment in sleep

Five participants changed their sleeping patterns to accommodate for fatigue. Four patients went to bed earlier to minimise the amount of fatigue felt after medication consumption. One patient mentioned that sleeping earlier also meant being able to wake early enough to fit in medications before work.

#### Changes in habits

Most participants changed their eating schedule (fasting an hour before and after medications, as instructed) and diet. Of those who smoked, one continued smoking at a reduced amount whilst all others quit. Fatigue resulted in a reduced ability to exercise in some patients.

### Video DOT

Video DOT patients preferred video DOT over clinic-based DOT, having found it convenient and very accessible. The video phone application was easy to learn and connection was generally good; one participant occasionally experienced slow connectivity.

“Coming every day to the hospital is definitely more difficult than learning to connect to a conference on whatever a laptop, tablet. I mean, it’s pretty flexible to you, you just need an electronic device”–Joseph, student

Half the video DOT patients mentioned that the fixed video appointment time was still hard to meet especially during work hours. Medication storage and organization at home was not an issue.

### Reducing treatment burden

Patients suggested two ways to reduce treatment burden: (1) better provision of health services and access and (2) a less burdensome treatment regimen.

#### Better provision of health services and access

Patients suggested improved access to medical assistance such as a direct line to a doctor when quick TB medical advice was required between scheduled doctor appointments. Some requested that chest clinic nurses were able to write medical certificates, instead of waiting for a scheduled doctor’s appointment. Other participants recommended more facilities for DOT (e.g. local GP) to reduce travel times. Having extended chest clinic hours was another suggestion to accommodate for those who had to work during opening hours.

Education was suggested to better equip patients for changes that treatment would have on their lifestyle and to inform society that TB is non-contagious when treated, to reduce social isolation of patients.

#### Less burdensome treatment regimens

Patients frequently suggested making medications easier to consume. Suggestions included reducing tablet size and number or introducing more liquid options. A participant on a combination of DOT and SAT found half tablets in his treatment regimen challenging, at times becoming confused about the number of tablets he was taking.

Two participants with multi-drug resistant TB suggested having a shorter treatment period. Three participants, two on video DOT and one on DOT, suggested recording medication consumption to be viewed by nurses later, such that direct observation is not in real-time. This would allow patients to complete treatment when free, rather than taking time out from work or study.

Nearly half (n = 9) of interviewees stated that SAT, either for part or all of treatment, would make treatment easier. Two participants did not support SAT, believing DOT is necessary to maintain compliance with treatment.

## Discussion

This study is the first to explore patient-perceived treatment burden in TB in a country with a low-incidence of TB where standard treatment involves clinic-based DOT and video DOT. This study has highlighted the substantial amount of time and effort required from TB patients to manage their treatment, similar to the treatment burden previously described in chronic conditions such as cystic fibrosis [18] and chronic obstructive pulmonary disease [19]. Participants described various forms of treatment burden (Fig 1) with the majority related to DOT and medication taking. Treatment burden had a significant impact on participants’ daily life and although many struggled with adjusting to a lengthy and time-consuming treatment regimen, most understood the importance of treatment completion.

Clinic-based DOT was physically and mentally draining to some patients. Key burdens included adjustment to personal life, work or study and direct and indirect costs, consistent with findings from a cross-sectional questionnaire study in Singapore [20] Some patients would have given up treatment if their regimen had not been altered from strict clinic-based DOT to include a combination of DOT and SAT, highlighting the high treatment burden associated with DOT. Importantly, DOT has not been shown to improve adherence to TB treatment or TB cure rates compared with SAT in a systematic review, when patients on SAT visited the TB clinic at least every 2 weeks [3].

An attempt to lower treatment burden and clinic non-attendance in countries with a high incidence of TB has given rise to community-based DOT—DOT provided in the community by community health care workers or community volunteers [21]. Patients find it more convenient, as treatment is delivered directly to their home and community healthcare providers are able to personally follow-up on delayed or missed doses [22].

Video DOT was positively regarded by most patients in this study. In other studies, treatment completion rates have been shown to be similar for video DOT and clinic-based DOT [23]. Video DOT provides flexibility and privacy to the patient, whilst saving time and direct costs [16]. The privacy aspect was important to patients who believed there to be perceived stigma about TB and were not comfortable being seen going to chest clinic. However, it requires a real-time cellular connection and is only feasible if patients have access to a suitable device and stable internet connection. Additionally, the nurses in our study setting reported that video DOT was more time consuming for them (as it required time to set up and sometimes required troubleshooting digital issues) than clinic-based DOT. Video DOT can be made more flexible for patients and health professionals by removing real-time direct observation, as suggested by several participants in our study. This method of video DOT, asynchronous video DOT, involves time-delayed observation of medication consumption through video uploads [24].

Difficulties with medication intake due to number or size of pills were another key burden identified, reflecting findings consistent with previous interview studies [8, 9]. In chronic conditions such as diabetes, fixed drug combination therapies have been found to improve patient satisfaction by combining multiple tablets into one, improving adherence to treatment [25]. Fixed drug combination therapy has been recommended by WHO for TB since 2001 through the combination of 2 to 4 anti-TB drugs [26]. Since then, several countries have adopted fixed drug combinations for TB treatment. However, these are not as flexible when patient-specific formulations are required, and the inseparable nature of the pill makes it difficult to determine the source of potential adverse effects. An increase in patient satisfaction when using fixed drug combinations has, however, been identified and the use of fixed dose combination therapy should be considered on a case-by-case basis, especially in those who struggle with swallowing tablets.

Alongside pill burden, treatment side effects had a significant impact on many patients, with some requiring extra medications to alleviate symptoms caused as a side effect of TB treatment, adding to their pill burden. Fatigue reduced quality of life and affected work and study. Fatigue was a substantial burden to many participants as it could not be alleviated by taking remedial medications. Patients who self-administered their medications at home had option to take the medications close to bedtime to alleviate the impact of fatigue on quality of life.

Some interviewees felt uncomfortable and stigmatised when attending the chest clinic for treatment. This, however, did not negatively impact treatment adherence in these interviewees, as described in a qualitative study in a high TB incidence setting [10]. Stigma is not just experienced by TB patients in settings with a high incidence of TB but also by patients in settings with a low incidence of TB [27], although the reasons might differ (e.g., TB may be linked to evil spirits in high incidence settings [28], whereas in low incidence setting stigma may be mainly the result of TB patients being considered infectious [even if they are not] [8] as experienced by some of our patients). Stigma can potentially be minimised through TB treatment delivery that is more private and flexible to patients, as previously discussed.

A strength of this study was the use of an iterative approach to allow interviews to be adapted in response to emergent themes. The semi-structured style of interviewing allowed participants to freely share their experiences. The study was conducted in a high-income country with a low TB incidence and findings from this study may not necessarily apply to low-income, high-incidence settings. However, although the severity of different areas of treatment burden may vary, most of the challenges experienced by our study participants will likely be similar across settings (e.g. loss of income due to treatment requirements) and probably more accentuated in low-income settings. It is possible that participants who agreed to an interview may have experienced more hardships with their treatment and thus were more willing to voice their dissatisfactions compared with patients who did not participate in the study.

## Conclusions

Our study highlights the substantial treatment burden associated with TB treatment, particularly with clinic-based DOT. Health providers should be aware of their patients’ capacity to handle treatment workload and that different burdens have different significance for individual patients. Measures to ameliorate the treatment burden include changes in treatment delivery on a case-by-case basis (for example, using approaches that combine DOT with self-administered therapy, video DOT (including asynchronous video DOT) and community-based DOT), providing better access to health services and improving patient education (before treatment is commenced) about the potential impact of TB treatment on patient’s everyday life. Development of a community-based DOT model using community workers or trained lay workers could potentially reduce patients’ treatment burden without overstretching health system resources.

In settings with a significant proportion of patients who prematurely cease TB treatment, research into the burden of treatment experienced by these patients may offer insights on how to improve health services to make them more patient-centred.

## Supporting information

S1 File Treatment burden in tuberculosis interview guide. (PDF)
