# Supplementary material for: Patient-perceived treatment burden of tuberculosis treatment
Source: PLoS One. 2020 Oct 22;15(10):e0241124. doi: 10.1371/journal.pone.0241124 (PMC7580887; doi:10.1371/journal.pone.0241124)
Supplement: S1 File — (PDF) [file pone.0241124.s001.pdf]

Patient-Perceived Treatment Burden of Tuberculosis Treatment

Authors: Natasha C.H. Ting, Nicole El-Turk, Michael S. H. Chou, Claudia C. Dobler

S1 File. Treatment Burden in Tuberculosis Interview Guide.

Question Domain	Main Question	Prompts
**Exploring burdens**	1. What do you know about tuberculosis? How do you know this?	• Did you have to look up your condition or ask someone about it?<br>• Did you have to Google 'tuberculosis'?<br>• Did you have to ask the doctors or nurses?<br>• Did family and friends have to explain it to you?
	2. What kind of things do you have to do for treatment?	• Take medications<br>• Plan your day
	3. Thinking of everything you have to do for your tuberculosis treatment, is there anything that affects your ability to manage the condition?	• Do you have school or work?<br>• Do you have to care for someone else and that gets in the way?
	4. Are there times where you find it difficult to do everything you need to do to get treatment? (**Link to healthcare burdens)**	
	5. Have you ever needed to cut back doing other things because of treatment?	• Have you ever needed to cut down on working?<br>• Have you ever felt like you can't do things you like anymore because of treatment?
	6. Do your treatments or self-care affect your social and family life? **(Link to social burdens)**	• Do you feel like you don't spend as much time with friends or family because of treatment?

	7. Has your treatment for tuberculosis affected you financially in any way? **If yes –** how has it affected you? **(Link to financial burdens)**	
**Lifestyle changes**	1. Have you needed to change your lifestyle to accommodate for the treatment you are receiving? **If yes** – what sort of changes?	• Have you ever needed to quit drinking alcohol, smoking, made diet changes etc.?
	2. Has your treatment restricted your ability to travel anywhere?	• Have you ever been advised against travelling overseas?<br>• Have you ever had to cancel overseas trips because of treatment?
**Healthcare burdens**	1. Have you struggled with keeping track of your appointments? **If yes** – is there a reason behind this?	• Have you ever forgotten about your appointments?
	2. Have you ever found it difficult to meet your scheduled appointment time? **If yes** – is there a reason behind this?	• Have you ever had to change appointments last minute because of other things e.g. work?
	3. Have you ever stopped your tuberculosis treatment before? **If yes** – could you tell me what made you stop and restart treatment again?	• Have you ever had issues with treatment that made you stop?
	4. Do you have to organise any medical paperwork, and if so, how are you managing with it?	• Do you ever have to obtain medical certificates?

	5. (If not previously raised) In terms of travel, how do you get to the clinic and how long does it take you	
	6. What is the hardest part about the travel, if you have any difficulties? How significant is this difficulty?	• Do you find the travel time too long?<br>• Is it stressful having to drive long distances?
	7. How is your relationship with the nurses and doctors? Are you comfortable asking them questions regarding your condition or treatment? **If no** – what is stopping you if you are comfortable sharing	
	8. What do you think would make your tuberculosis treatment a lot easier for you?	• For example, providing parking for you<br>• For example, sending a reminder text the day before appointments<br><br>(Guide towards approaches that we can actually change)
**Medication burdens**	1. How often do you receive directly observed therapy and how much time do you have to spend each visit?	• Do you think this amount of time is too long?<br>• How much time is spent waiting for treatment or for an appointment?
	2. Have you had any side-effects from the treatment that you needed additional treatment? **If yes** – what additional treatment has been required	
	3. Do you have any difficulties with taking the medications itself e.g. swallowing tablets	• What in particular is hard about taking the treatment e.g. taste, number of pills, pill size etc.

	4. **For those on video directly observed treatment (VDOT)** – How are you managing your medications at home and organising your medication intake? Do you feel well-equipped and supported? **If no** – why and what could be done to make things easier	
	5. **For those on video directly observed treatment (VDOT)** – how difficult was it to install and learn how to use the app? Have you had issues with VDOT?	• Have you had issues with connectivity?<br>• Have you not been able to digitally connect with the nurse supervising?
	6. **For those on video directly observed treatment (VDOT)** – would you recommend this video method to other patients? Why or why not?	
**Social burdens**	1. Have you ever felt isolated from those around you due to your condition? **If yes** – how has the isolation affected you and has it prevented you from continuing your treatment	• Have you not had time to spend with family and friends because of appointments?<br>• Have you felt worried about side effects and so avoid going out/socialising?
	2. Do you require someone else to help you with your tuberculosis care? **If yes** – who helps you, what do they do to help you?	
	3. Have you ever felt stigmatised (i.e. looked at or treated differently after your diagnosis)? **If yes** – can you give me an example of a time when you felt stigmatised	• Do people treat you differently when they know you are receiving treatment for tuberculosis?
	4. How has your treatment affected your working/studying life?	• Does it disrupt your study schedule?<br>• Does it become hard to do the work you need to do?<br>• Do you feel tired after taking the medications?

		• Do you feel like the treatment makes you tired and so this affects how well you work?
	4. (For those who are studying or employed) Do you think your treatment affects the way your colleagues or employer views you? **If yes** – do feel as if it has had a negative consequence? Give an example if so.	
**Financial burdens**	1. Do you have to take time off work to visit the clinic? **If yes** – how long do you usually take off work and has it had any consequences on your career?	• Does treatment make you tired after and affect your work in this way?
	2. What other things do you have to pay for in order to get treatment (acknowledge that the treatment itself is free)	• Do you have to pay for transport?<br>• Do you have to pay for parking?<br>• How much would you have spent on treatment related costs so far?
	3. Do you feel like your treatment is a burden financially?	• Do you find it expensive paying for public transport each time?
